# *Pneumocystis*: Where Does It Live?

**DOI:** 10.1371/journal.ppat.1003025

**Published:** 2012-11-29

**Authors:** Francis Gigliotti, Terry W. Wright

**Affiliations:** Division of Pediatric Infectious Diseases, Department of Pediatrics, University of Rochester School of Medicine, Rochester, New York, United States of America; Duke University Medical Center, United States of America

*Pneumocystis* is an unusual fungus that is a prototypical opportunistic pathogen, causing an asymptomatic or mild infection in the normal host but fulminate pneumonia (PcP) in the immunocompromised host. Untreated, the mortality rate from PcP approaches 100%. Even with treatment, mortality rates approach 10–20%. It is a ubiquitous organism infecting a wide array of mammalian species. Although the reservoir of infection for *Pneumocystis* has not been defined, direct airborne transmission from host to host has been proven under experimental conditions using the rat model of PcP [1]. This synopsis will review the evidence suggesting that the reservoir of infection for humans with PcP is other humans, possibly infants and young children.

The study of *Pneumocystis* has been problematic due to the inability to cultivate the organism or manipulate its cellular or molecular characteristics. As recently as the 1970s, a student studying *Pneumocystis* would have come away with the following understanding of its basic biology and function as a pathogen: *Pneumocystis* is an organism of low virulence found in many mammalian species. In humans, *Pneumocystis* pneumonia (PcP) is a zoonosis resulting from reactivation of a latent infection acquired early in life. This concept of *Pneumocystis* arose largely through analogy to existing knowledge about other organisms to explain clinical observations, rather than through direct experimentation on the organism. Over the past 25 years, we have learned more about *Pneumocystis* through controlled studies that have corrected some of the misconceptions contained in the “old” concept of *Pneumocystis* contagion stated above. What follows is a brief summary of key research observations that give us a better, yet still incomplete, understanding of how *Pneumocystis* maintains its existence as an opportunistic pathogen.

## *Pneumocystis* Is Not a Zoonosis

In order for *Pneumocystis* to be classified a zoonosis it would need to be transmissible from one host species to another. This was originally suspected for *Pneumocystis* because human and rodent *Pneumocystis* appeared visually similar following histochemical staining procedures. However with the advent of monoclonal antibodies, it was possible to show that *Pneumocystis* from one host species was phenotypically distinct from *Pneumocystis* from a different host [2]. Soon thereafter this phenotypic variation was confirmed at the genetic level [3]–[5].

Experiments that directly assessed the ability of *Pneumocystis* to move between species were published in 1993. This study demonstrated that *Pneumocystis* could not be transmitted between mice and ferrets [6]. This observation was soon followed by similar results that showed *Pneumocystis* from rabbits, rats, monkeys, and humans were not infectious for mice [reviewed in 7]. Thus, available data indicates that each mammalian species that contracts PcP has its own strain or species of *Pneumocystis*.

## *Pneumocystis* Does Not Establish Latency under Laboratory Conditions

To experimentally address the issue of latency, severe combined immunodeficiency disease (scid) mice were allowed to develop active PcP prior to restoration of their immune system by adoptive transfer of normal spleen cells. Immune-reconstituted mice recovered from PcP through an immune response without the use of antibiotics. *Pneumocystis* was cleared from the lung and could not be detected by three weeks post-reconstitution. To determine if the mice remained latently infected, they were again immunosuppressed and observed for up to 84 days for reactivation of PcP, which did not occur [8]. Similar results, using a somewhat different approach in rats, were obtained by Vargas et al. [9]. Thus, normal immune mechanisms are sufficient to clear *Pneumocystis* after infection such that latency is unlikely.

Support for the concept that PcP in adults is a result of recent acquisition of *Pneumocystis*, rather than reactivation of a latent infection, comes from analysis of second episodes of PcP. Keely et al. were able to show that episodes of PcP occurring more than six months apart were caused by genetically distinct strains of *Pneumocystis* [10]. Furthermore, genetic typing of *Pneumocystis* isolates from human patients demonstrated that the genotype of the organism causing disease was associated with the patient's place of residence rather than place of birth [11].

## *Pneumocystis* Infects the Normal Host, Producing a Typical Pattern of Contagion Followed by Immune Response and Clearance

To characterize the course of infection in the normal host, we exposed immunocompetent mice to scid mice with active PcP [12]. The normal mice developed a productive infection with growth of organisms through week four, at which time *Pneumocystis*-specific antibodies appeared in the serum in association with complete clearance of organisms. However, normal mice with this self-limited mild infection were able to transmit *Pneumocystis* to another normal mouse, thereby serving to maintain *Pneumocystis* in the environment. Alternatively, if the infected normal mouse encountered an immunosuppressed host, full-blown PcP resulted [13], [14]. Thus the normal host could serve as a reservoir to maintain *Pneumocystis* in any given mammalian population.

## Asymptomatic or Subclinical Infection of Human Infants is Common

It has been long known that low titers of antibody to *Pneumocystis* are common in humans. The role of young children in the contagion cycle was suggested by the observation that *Pneumocystis* was frequently found in the lungs of infants dying of Sudden Infant Death Syndrome (SIDS) in Santiago, Chile; Oxford, England; Rochester, New York; and Connecticut [15], [16]. While it was initially postulated that *Pneumocystis* was somehow contributing to SIDS, examination of the lung histology of infants dying of SIDS and who were also infected with *Pneumocystis* revealed very few organisms and no evidence of an inflammatory reaction. Additional studies do not support any causative role for *Pneumocystis* in SIDS [17]. They do, however, support the concept that infection of infants is common. These findings are consistent with the findings reported for normal mice infected with *Pneumocystis* discussed above and are also consistent with studies by Garvy et al., which demonstrate that neonatal mice are highly susceptible to infection with *Pneumocystis* [18].

To better understand the kinetics of infection, we carried out a prospective study of primary infection in 107 infants in Santiago, Chile. Infants were tested for the presence of serum antibody to *Pneumocystis* at birth and every two months thereafter for two years. Nasopharyngeal aspirates were also examined for *Pneumocystis* by PCR during respiratory illnesses. At two months of age, 16% were seropositive and seroconversion occurred at a rate of approximately 5% per month. By 20 months of age, 85% of the cohort of infants had seroconverted. Seventy-four infants were screened for *Pneumocystis* DNA and 32% were positive [19]. Given that the peak age for SIDS overlaps with the peak time of seroconversion, the presence of *Pneumocystis* in the lungs of SIDS cases is likely coincidental.

## Infants and Children Are a *Pneumocystis*-Susceptible Population That Could Serve as an Important Reservoir for Maintaining *Pneumocystis* in the Environment

**Figure 1:**
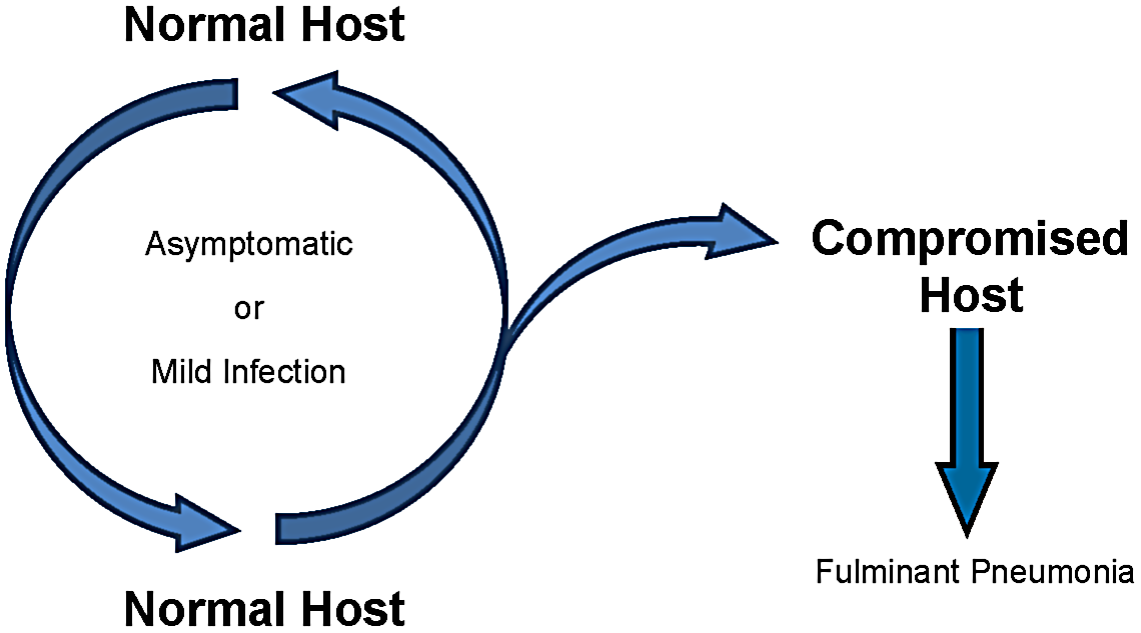
Proposed transmission cycle for *Pneumocystis* based on the normal host, likely infants, serving as the reservoir of infection.

By extending the experimental observations made in animal models of PcP to the epidemiologic observations in infants, one can envision that infants serve to harbor *Pneumocystis* with little adverse effect on the host. Once infected, an infant could then pass on the infection to another host, either normal or immunocompromised. Resistance to reinfection is of unknown duration but would be compensated for by new births that would serve to maintain a pool of susceptible hosts. As depicted in Figure 1, circulation in the healthy population would result in subclinical or mild infection. It is quite possible that one of the multitude of “viral illnesses” occurring within the first two years of life is actually an infection with *Pneumocystis*. When an immunocompromised host enters the cycle, the result is active PcP. The compromised host can then spread infection to a compromised patient, as has been suggested by clinic “outbreaks” [20], or back to a normal individual where it can maintain a quasi-symbiotic relationship of limited duration with its human host. Studies have not been done to determine the frequency with which normal older children and adults become infected, so we don't know whether older children and adults also serve as a significant reservoir of infection. However, *Pneumocystis* DNA has been found in oral washes from adult patients [21].

So where does *Pneumocystis* live? Given what we have learned in the past two decades, the most likely answer to this question is: It lives in us. Our concept of *Pneumocystis* should now read: *Pneumocystis* is a classic opportunistic pathogen that causes little or no disease after infecting a normal host, while infection of the immunocompromised host results in a uniformly fatal pneumonia if untreated. Each susceptible mammalian species is infected by a unique strain or species of *Pneumocystis*, but the basis for the host-species specificity remains a mystery. In man, the organism is spread from person to person, and PcP is most likely the result of recent acquisition of *Pneumocystis* rather than reactivation of a latent infection. The ubiquitous presence of *Pneumocystis* throughout the population likely explains why this organism is so adept at “finding” immunocompromised patients and suggests that more widespread use of immunomodulatory therapies will increase the risk of developing PcP. Understanding this aspect of the biology of *Pneumocystis* will aid in developing means to prevent PcP.
